# Contribution to the History of Malaria

**Published:** 1845-03

**Authors:** R. S. Holmes

**Affiliations:** Med. Staff U. S. A.; Philadelphia


*Contribution to the History of Malaria. In a letter from* Dr. R. S. Holmes, *of the Medical Staff of the United States Army, to* Professor Dunglison. (Communicated by the latter.)

*Philadelphia*, November 23d, 1844.

To Professor Dunglison:

Sir,—You were so kind as to call my attention, lately, to an extract from a letter written by me, dated "Fort Macomb, Middle Florida," which you did me the honour to insert in the last edition of your work on Human Health, or the Elements of Hygiène, in which I mentioned the great difficulty I experienced in accounting for the unhealthy character of that Post, it being situated on a bluff, above the River Suwannee, out of the neighbourhood of marshes,—a lofty growth of pines extending for many miles back from the Post, and the sand on which it was built, and around it, being of that pure white character so common to Florida. I also hinted at my suspicions of some geological cause being at work in the production of disease; and I am happy to say, further investigations confirmed these views.

In a Report on the Medical Topography of the Post made for the Surgeon General a few weeks after I had been stationed there, I expressed my utter inability to account for the volumes of miasmata (known by its effects on nearly every man in the command) that were generated around us. In a subsequent Report, made after four months sojourn there, I accounted for it, at least entirely to my own satisfaction, in the following manner.

During a tedious convalescence from a low type of congestive fever, my short walk of some forty or fifty yards from the Post was always terminated by a profuse growth of fungi, of a single species, known to botanists as the *phyllus fœtidus.* This locality, being the principal one in the neighbourhood, covered a space of about two hundred square yards. Why the walk terminated here will be perfectly evident to any one acquainted with the vegetable growth in question: there is probably no living vegetable that can and does exhale so disgusting an odour as this fungus. If it is in its *living* state that this odour is exhaled, or only in its *decay*, I never had the hardihood to ascertain; for as the fungus lasts only for twenty-four or thirty hours, and there is a new growth every morning, the dead plant is always present; neither am I certain, nor do I see any reason, why this fungus, in its decay, which commences some ten or fifteen hours after it appears above the ground, may not produce disease. I certainly have seen from three to four hundred of these most beautiful but disgusting vegetables, dotting with their pure vermillion colour nearly every inch of ground in the locality. I will not stop, however, to inquire about their power in disease, but from their potency on the organs of smelling, one could readily believe that they grew among Milton's,

"Rocks, caves, dens, pools, swamps, bogs and shades of death!"

But as this was in the autumn and winter months, when rain does not fall in Florida, often for many weeks, and this season especially offered a constant succession of bright, clear days; and as the fungus requires an immediate supply of moisture for its growth, it became an important question to know how this moisture was obtained. The locality was on a level patch of sandy soil, fifty feet above the river, and not one hundred yards from it; at one side there was a gentle descent toward a small stream entering into the Suwannee; so that the spot on which the growth was most abundant, was really on an elevation. I had the earth dug thereabouts; and at depths varying from one to several feet, I found a stratum of white stiff clay, perfectly impervious to water infiltrated through the sand above it, and retaining it on its surface as effectually as if it had been a coating of india rubber spread beneath.

I thought I thus obtained the solution of the mystery; the Fort had been selected on account of its apparently healthy situation ; and the cause of disease had been a puzzle to many others beside myself. I found this stratum of clay extended for several hundred yards in certain directions; you might trace it with almost unerring certainty by the nature of the dead and living vegetable substances on the sand above it. Heavy branches and trunks of pines, and oaks, would be moist and wet in the dry months of winter in Florida, while a profuse growth of ephemeral mushrooms, such as spring up in the North only after heavy rains, would meet the eye at every step, even when rain had not fallen for several weeks.

Any one who knows the nature of the forests of Florida will see how the constant presence of moisture among the great number of branches, leaves, and trunks of trees that cover the ground, will be a fruitful cause of decay among them, taking place as speedily as the nature of the wood will permit; for, unlike the forest trees of the North, those of the South, from the greater amount of resinous principles in them, resist decay for a much longer time.

Very truly and respectfully,
Your obedient servant,
R. S. Holmes,
Med. Staff U. S. A.

